# Serratus Anterior Fascia Free Flap for Functional Surgery of Subungual Melanoma: Case Series and Literature Review

**DOI:** 10.1055/s-0044-1792109

**Published:** 2025-03-11

**Authors:** Soo Jin Woo, Sung Tack Kwon, Byung Jun Kim

**Affiliations:** 1 Department of Plastic and Reconstructive Surgery, Seoul National University College of Medicine, Seoul, Republic of Korea

**Keywords:** subungual melanoma, serratus anterior fascia free flap, nail unit, fingertip, microsurgery

## Abstract

**Background** In addressing subungual melanoma, this study presents the efficacy of wide excision followed by reconstruction using a serratus anterior fascial free flap.

**Methods** The study covers four patients treated between 2017 and 2020 for melanoma in the great toe or thumb, highlighting the successful application of the flap and split-thickness skin graft over exposed distal phalanx cortical bone.

**Results** The Breslow depths of the melanomas ranged from 0.2 to 6 mm, with four to seven lymph nodes dissected per patient, revealing no metastasis. Over follow-ups of 26 to 57 months, no local or distant recurrences were observed. The serratus anterior fascial free flaps, averaging 2.1 mm in thickness, precisely matched defect depths, negating the need for further debulking.

**Conclusion** This technique offered satisfactory functional and aesthetic outcomes, proposing the serratus anterior fascial free flap as a viable alternative for acral region reconstruction in subungual melanoma cases after wide excision.

## Introduction

Malignant melanoma, representing less than 5% of skin cancers, is the leading cause of skin cancer deaths. 1 Subungual melanoma (SUM), a rare subtype found in nail units, comprises about 1 to 3% of melanomas, 2 with a higher prevalence in dark-skinned populations. 3 Traditionally treated with amputation, recent evidence suggests wide excision offers similar outcomes without compromising function and aesthetics. 4 5 While skin grafts are common, they may contract and disfigure, especially over bony defects. Alternative regional flaps are limited by donor site availability and morbidity. The serratus anterior fascial free flap (SAFFF), a thin, easily moldable option, presents a solution without the need for debulking. This case series evaluates the efficacy of SAFFF in four patients with SUM following wide excision, underscoring its potential as a reconstructive technique. Additionally, we conducted a literature review of journals that have utilized flaps for the reconstruction of SUM.

## Case

The cohort comprised four female patients aged 45 to 81 years, treated for SUM with SAFFF reconstructions involving two great toes and two thumbs. The Breslow depths ranged from 0.2 to 6 mm ( Table 1 ). One patient (case 4) uniquely declined amputation, opting for conservative management despite not meeting the criteria for functional surgery and presenting a metastatic risk. Preoperative evaluation confirmed primary malignant melanoma without distant metastasis in all patients.

**Table 1:** Demographic and clinical characteristics of the four study participants

Case	Sex	Age (y)	Location	Breslow's depth (mm)	Stage (based on the 8th edition of AJCC)		Defect volume (cm × cm × cm)	Flap size (cm)	Flap thickness (mm)	Pedicle length (cm)	Follow-up period (mo)	Two-point discrimination test (mm)	Range of motion in interphalangeal joint (flexion /extension)	Satisfaction score
1	F	81	Right great toe	2.5	T3bN0M0	IIB	4.0 × 4.0 × 2.1	4 × 5	2.2	3.1	57	7.8	75/–10 degrees	Good
2	F	49	Right thumb	0.2	TisN0M0	MIS	3.4 × 2.9 × 1.4	7 × 6	1.8	4.0	42	7.0	60/0 degrees	Fair
3	F	45	Right great toe	2	T2bN0M0	IIA	3.1 × 2.7 × 1.1	4 × 2.5	2.0	3.5	41	8.0	85/10 degrees	Excellent
4	F	56	Left thumb	6	T4bN0M0	IIC	3.9 × 3.2 × 1.5	3 × 4.5	2.4	3.7	26	6.5	65/10 degrees	Excellent

All surgeries were performed by two teams. The patient was placed in the lateral decubitus position, and one team performed wide excision of the SUM, while another team elevated the SAFFF on the contralateral side of the patient. Functional wide excision of the SUM was performed with a 5- to 10-mm safety margin around the nail plate border or pigmented lesion. The nail apparatus and periungual soft tissue, including the periosteum over the bony cortex of the distal phalanx, were resected en bloc. According to the National Comprehensive Cancer Network guidelines, sentinel lymph node dissection should be performed for lesions with a Breslow depth over 1 mm or lesions with other adverse features (e.g., mitotic index >2/mm ^2^ , particularly in young patients, lymphovascular invasion, or a combination of these factors).

**Fig. 1:**
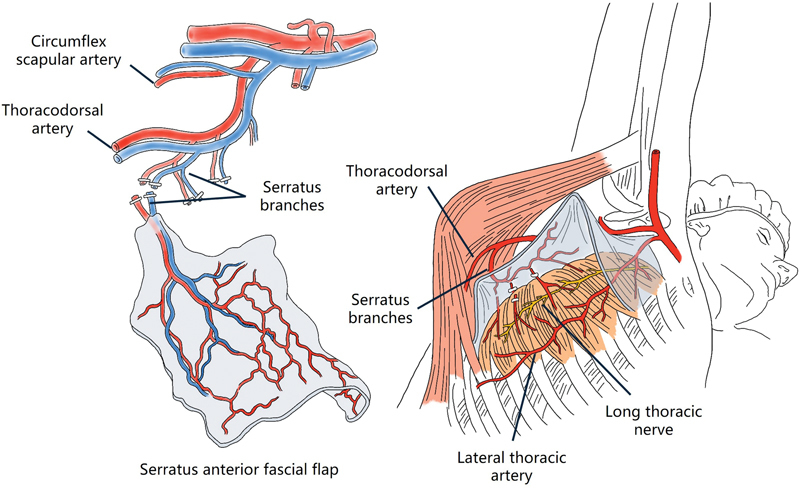
Schematic drawing of serratus anterior fascial flap.

The SAFFF was harvested with a lazy **S** -shaped incision (10 cm) beginning at the axilla, near the anterior border of the latissimus dorsi muscle. The latissimus dorsi muscle was retracted posteriorly, and the serratus anterior muscle and serratus anterior branch vessel were identified ( Fig. 1 ). The pedicle was dissected proximally up to the main thoracodorsal vessel. Care was taken to preserve the long thoracic nerve during the separation of the serratus anterior fascia from the serratus anterior muscle. The digital artery and dorsal superficial vein of the dominant side were used as recipient vessels. The fascial flap was positioned over the defect with the deep side of the flap facing superficially. Microanastomosis was performed with 10–0 nylon, and the flap was covered with a split-thickness skin graft (STSG) measuring 10/1,000 inches, harvested from the lateral thigh. Wound dressing was performed carefully to ensure that no unnecessary pressure was applied to the pedicle of the acral area. A splint was applied for 2 weeks postoperatively.

The pedicle length and flap thickness were measured at the center near the pedicle. Postoperatively, the range of motion (ROM) at the interphalangeal joint (IPJ) and the values of two-point discrimination (2PD) tests were assessed during the patients' visit at 18 months after surgery by the surgeon. Aesthetic satisfaction scores were assessed subjectively by the patients. An “excellent” result was defined as a finger or toe with soft tissue volume similar to the contralateral digit. A “good” result was a slight volume discrepancy noticeable only to the patient. A “fair” result was a volume discrepancy noticeable to others, and a “poor” result was defined by an unacceptable appearance.

### Results

**Fig. 2:**
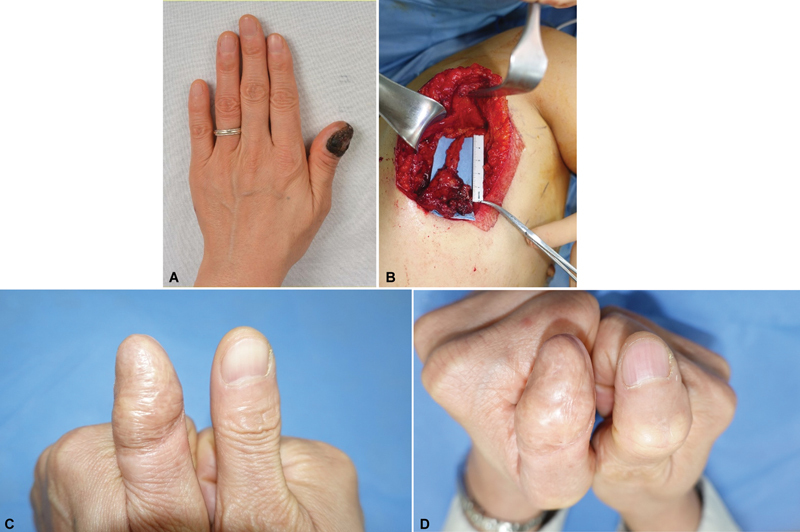
Clinical photographs of the fourth patient. ( **A** ) A 56-year-old woman with a subungual melanoma of the left thumb. ( **B** ) Intraoperative view of the elevated serratus anterior fascial free flap. ( **C, D** ) Two-year postoperative photographs showing good aesthetic results with normal interphalangeal joint motion.

The flap thickness averaged 2.1 mm (range, 1.8–2.4 mm) and the pedicle length averaged 3.6 cm (range, 3.1–4 cm; Table 1 ). The follow-up period averaged 41.5 months, with a range from 26 to 57 months, during which no recurrences were observed. All flaps demonstrated complete survival, and no donor site complications were noted. Patients returned to their normal daily activities 2 weeks postoperatively. The 2PD test results ranged from 6.5 to 8.0 mm. The mean ROM of the IPJ included a flexion of 72 degrees (range, 60–85 degrees) and an extension of 2.5 degrees (range, −10 to 10 degrees). Patient satisfaction scores were reported as excellent by two patients, good by one patient, and fair by one patient ( Fig. 2 ).

## Discussion

In treating SUM, the traditional approach of digit amputation is being reconsidered in favor of limb-sparing techniques such as wide excision, which recent evidence suggests offers similar prognostic outcomes without functional and aesthetic compromise. 4 This shift aligns with our findings, where the SAFFF effectively reconstructed acral skin defects following SUM excision, emphasizing the trend toward preserving digit integrity and function.

Skin grafting is commonly used to reconstruct extensive defects of the acral skin. While it is technically simple, 6 graft failure is also common, particularly when the primary defect is large or involves bone. Patients may also complain of increased sensitivity to cold or trauma. 5 Other local flaps, such as the heterodigital island flap 7 or cross-finger flap, 8 have been discussed because these obviate the need for microanastomosis. However, the hand offers a limited amount of viable soft tissue for skin flaps, and short pedicle lengths increase the likelihood for secondary surgery and donor site complications, such as immobilization. 9 Recently, significant efforts have been directed toward achieving the thinnest possible flaps for reconstruction. 10 11 12 Several thin free flaps have been explored for reconstruction following functional surgery of SUM ( Table 2 ). 13 14 15 16 These flaps share a common advantage that they offer excellent aesthetic outcomes in addition to functional benefits following digit-sparing surgical approaches.

**Table 2:** Review of free flap approaches following functional surgery for subungual melanoma

Study	No. of patients	Localization	Breslow's thickness (mm)	Type of defect	Type of free flap	Flap thickness	Oncologic outcomes	Functional outcomes	Aesthetic outcomes
Motta et al 13	1	Thumb	–	Wide local excision	Onychocutaneous toe free flap	–	No recurrence at the 3-mo follow-up	Full range of motion of IPJ	Normal nail growth
Lee et al 14	40	Thumb: 17 Index finger: 3 Middle finger: 2 Little finger 2 Great toe: 14 2nd toe: 1 3rd toe: 1	0.67 (0–3)	Wide local excision	Super-thin free superficial circumflex iliac artery perforator flap	4 (3–8) and 1.5–4 mm after primary defatting	Two recurrences, one local recurrence and one in-transit recurrence at the 31-mo follow-up 3-y local recurrence free: 97.1% 3-y disease-free survival: 97.1%	Quick-DASH score: 1.3 (0–6.8) Foot function index: 3.1 (0–8.0)	Majority of patients achieved satisfactory contour
Woo et al 15	17	Thumb: 7 Index finger: 1 Long finger: 3 Ring finger: 1 Great toe: 5	1.2 ± 1.1 (0–4)	Wide local excision	Arterialized venous free flap	One case underwent secondary debulking procedure	Three local recurrences, one distant metastasis after 75.5 mo of follow-up	*Subjective assessment* Excellent (10) Fail (4)	*Subjective assessment* Excellent (8) Fail (4) Poor (2)
Kim and Lee 16	2	Thumb: 1 Ring finger: 1	0	Circumferential defect around bone	Superficial palmar branch of the radial artery flap	–	No recurrence at 24 mo of follow-up	Full range of motion of the IPJ	*Subjective assessment* Fair (1) Good (1)
Current study	4	Thumb: 2 Great toe: 2	2.7 (0.2–6)	Wide local excision	Serratus anterior fascia free flap	2.1 (1.8–2.4) mm	No recurrence at 41.5 mo of follow-up	2PD test: 7.3 (6.5–8.0) mm IPJ flexion: 72 (60–85) degrees IPJ extension: 2.5 (−10 to 10) degrees	*Subjective assessment* Excellent (2) Good (1) Fair (1)

Abbreviation: 2PD, two-point discrimination; IPJ, interphalangeal joint; QuickDASH, Quick Disabilities of the Arm, Shoulder, and Hand.

This study is the first to examine the SAFFF's role in reconstructing acral skin defects following functional surgery for SUM. Previously, the SAFFF was used for reconstructing hand dorsal surface defects due to its pliability and suitability for extensor tendon movement. Identified by Wintsch and Helaly as loose connective tissue between the latissimus dorsi and serratus lateralis muscles, this “gliding tissue flap” was recommended for reconstructing adherent tendons or defects on the hand and foot dorsum. 17 18

The advantage of the SAFFF in this procedure is that it can be harvested by two teams without repositioning the patient intraoperatively. Additionally, the flap can be easily tailored to match the required dimensions, and the donor site heals with a thin, linear scar without functional deficits. Another significant benefit was the flap's thickness closely matching the defect depth. The distance from the eponychium to the underlying distal phalanx bony cortex was 1.91 ± 0.49 mm at the thumb and 2.08 ± 0.49 mm at the big toe. 19 The SAFFF's mean thickness of approximately 2.1 mm provided a natural-looking fingertip contour without the need for additional debulking surgery. However, potential drawbacks include the rare but significant risk of injury to the long thoracic nerve during harvesting. After flap inset, an additional skin graft might be required for coverage, which could be delayed if persistent oozing occurs. Monitoring a fascial flap under a skin graft is challenging, potentially delaying the detection of vascular issues.

This study underscores the SAFFF's successful application for acral skin reconstruction after wide excision of SUM. It was advantageous in matching the nail bed defect depth and maintaining the digit's natural contour. The outcomes were significant for providing adequate soft tissue coverage, preserving the digit tip's anatomical integrity, and minimal donor site morbidity. These findings suggest the SAFFF as a valuable alternative in SUM management, offering notable benefits in tissue integration and aesthetic outcomes with minimal donor site morbidity.
